# Deletion of the Response Regulator PhoP Accelerates the Formation of Aerial Mycelium and Spores in *Actinosynnema pretiosum*

**DOI:** 10.3389/fmicb.2022.845620

**Published:** 2022-04-06

**Authors:** Peipei Zhang, Kunyu Zhang, Yayu Liu, Jiafang Fu, Gongli Zong, Xin Ma, Guangxiang Cao

**Affiliations:** ^1^Department of Epidemiology, The First Affiliated Hospital of Shandong First Medical University, Jinan, China; ^2^College of Biomedical Sciences, Shandong First Medical University and Shandong Academy of Medical Sciences, Jinan, China

**Keywords:** *Actinosynnema pretiosum*, morphological development, PhoP, PHO box, TCS

## Abstract

PhoPR is an important two-component signal transduction system (TCS) for microorganisms to sense and respond to phosphate limitation. Although the response regulator PhoP controls morphological development and secondary metabolism in various *Streptomyces* species, the function of PhoP in *Actinosynnema pretiosum* remains unclear. In this study, we showed that PhoP significantly represses the morphological development of the *A. pretiosum* X47 strain. Production of aerial mycelium and spore formation occurred much earlier in the ΔphoP strain than in X47 during growth on ISP2 medium. Transcription analysis indicated that 222 genes were differentially expressed in ∆phoP compared to strain X47. Chemotaxis genes (*cheA*, *cheW*, *cheX*, and *cheY*); flagellum biosynthesis and motility genes (*flgBCDGKLN*, *flaD*, *fliD-R*, *motA*, and *swrD*); and differentiation genes (*whiB* and *ssgB*) were significantly upregulated in ∆phoP. Gel-shift analysis indicated that PhoP binds to the promoters of *flgB*, *flaD*, and *ssgB* genes, and PHO box-like motif with the 8-bp conserved sequence GTTCACGC was identified. The transcription of *phoP*/*phoR* of X47 strain was induced at low phosphate concentration. Our results demonstrate that PhoP is a negative regulator that controls the morphological development of *A. pretiosum* X47 by repressing the transcription of differentiation genes.

## Introduction

*Actinosynnema pretiosum* is a Gram-positive, filamentous bacterium that is characterized by motile spores and the ability to produce ansamitocin P-3 (AP-3), a potential anti-tumor agent (Yu et al., 2002; Martin et al., 2014). Although AP-3 has commercial value, it is produced at low levels, and therefore, it is of interest to increase its production by *A. pretiosum* (Li et al., 2016; Liu et al., 2020; Wu et al., 2020; Wang et al., 2021). *Actinosynnema pretiosum* X47 in this study was derived from *A. pretiosum* subspecies *auranticum* ATCC 31565. The genome of *A. pretiosum* X47 was about 8.13 Mb in length, with an average GC content of 73.91%, and 7029 genes were predicted, including 45 pairs of putative two-component signal transduction system (TCS), 13 histidine kinases, and 38 orphan response regulators (Zhong et al., 2019). The morphological development of *A. pretiosum* is complex and similar to that of *Streptomyces*. *Actinosynnema pretiosum* strains can form branched vegetative hyphae and aerial hyphae on solid media, and the aerial hyphae can separate into chains of spores, which are motile in liquid medium due to flagella (Hasegawa, 1983).

TSCs consist of a histidine kinase (HK), sensing environmental changes or nutrition limitation, and a response regulator (RR) responding to these changes *via* the modification of the expression of specific genes. PhoPR is an important TCS controlling the adaptation of the metabolism to phosphorus limitation. PhoR is the HK of the PhoPR system and has a transmembrane region; PhoP is the RR and belongs to the OmpR family of regulators (Galperin, 2010). In *Mycobacterium tuberculosis*, PhoPR is essential for growth and virulence. In a deletion mutant of *phoP*, the synthesis of the cell envelope and the growth of the *M. tuberculosis* H37Rv strain is inhibit in host systems (Perez et al., 2001; Walters et al., 2006; Ryndak et al., 2008; Broset et al., 2015). PhoPR also plays a crucial role in the virulence and pathogenicity of *Corynebacterium pseudotuberculosis* (Tiwari et al., 2014). In *Streptomyces*, PhoP serves as a global regulator that influences morphological development and antibiotic synthesis (Martin et al., 2012, 2017; Yang et al., 2015). PhoP represses the expression of differentiation genes, including *bldA*, *bldC*, *bldD*, and *whiH*, which influence the growth of *Streptomyces coelicolor* (Ryding et al., 1998; den Hengst et al., 2010; Hackl and Bechthold, 2015; Schumacher et al., 2018), and PhoP activates the biosynthesis of the secondary metabolites actinorhodin and undecylprodigiosin (Rodriguez-Garcia et al., 2007; Martin et al., 2017). Deletion of *phoP* resulted in poor growth of *Streptomyces avermitilis* on soya flour mannitol medium without phosphate (Pi) supplements but significantly increased avermectin biosynthesis; however, the growth of the *phoP* deletion mutant and wild-type strains was similar when Pi was added in the medium (Yang et al., 2015; Martin et al., 2017). Similar results were also obtained with *Streptomyces lividans* (Smirnov et al., 2015). In *Streptomyces filipinensis*, growth of the *phoP* mutant strain was greatly affected and it formed fewer spores than the wild-type strain upon growth on a Pi limited medium, whereas it yielded far more abundant spores than the latter on a Pi proficient medium (Barreales et al., 2018).

Although PhoPR is essential for the normal growth and metabolism of a broad range of species, the regulatory functions and mechanisms of PhoP differ in different bacteria. In this study, the PhoPR homolog was identified in the genome of the *A. pretiosum* X47 strain (Zhong et al., 2019), and a *phoP* mutant strain was constructed. Our data suggest that deletion of *phoP* accelerates the formation of aerial mycelia and spore formation in strain X47, contributing to a better understanding of developmental regulation in *A. pretiosum* strains.

## Materials and Methods

### Bacterial Strains and Culture Conditions

*Actinosynnema pretiosum* X47 and its derivatives were cultured at 30°C on solid ISP2 medium (Ma et al., 2007) for spore production and conjugation. *Escherichia coli* strains DH5α, BL21, and ET12567 (pUZ8002; Kieser, 2000) were grown in Luria-Bertani (LB) medium or on LB agar at 37°C, for genetic engineering, protein expression, and conjugation, respectively. Antibiotics were used to select genetically modified *A. pretiosum* and *E. coli* strains*. A. pretiosum* is sensitive to apramycin and hygromycin B. Apramycin is used to screen mutant strains of *A. pretiosum* and hygromycin B is for complementary strain screening.

### Deletion of the *phoP* Gene From the Strain *Actinosynnema pretiosum* X47

To construct the *phoP* mutant strain ΔphoP, flanking sequences of approximately 1,500 bp from the left and right sides of *phoP* were amplified from the X47 genome using primers PhoP-L-F/L-R and PhoP-R-F/R-R (Supplementary Table S1). An apramycin resistance cassette sequence was amplified from pSET152 (Bierman et al., 1992) using primers Apra-F and Apra-R. Then, the left arm, resistance cassette, and right arm were ligated and inserted into pMD18-T (TaKaRa) using the ClonExpress II One Step Cloning Kit. Subsequently, the fragment constituted by the left arm, resistance cassette, and right arm was released by digestion with *Xba*I and *Hind*III and inserted into pJTU1278 (He et al., 2010) to generate plasmid pM-phoP. The resulting plasmid was then transferred into *E. coli* ET12567 (pUZ8002), and the transformants were used as donors to conjugate with *A. pretiosum* X47, as described by Kieser (2000). Apramycin-resistant conjugates were selected and the genomic structure of the ΔphoP strains was verified by PCR with the primers PhoP-V-F/R. The phenotypes of the strains were observed when cultured on solid ISP2 medium.

### Complementation of ΔphoP

To complement the *phoP* deletion mutant, a 2,270-bp fragment containing the coding region of *phoP* and *phoR* and a 300-bp region upstream of *phoR* was amplified from the X47 genome using primers PhoP-Com-F/R. The PCR product was inserted into pMD-18T and cloned as a *Hind* III fragment into pMS82, resulting in plasmid pC-phoP. Plasmid pC-phoP was transformed into *E. coli* ET12567 (pUZ8002), and hygromycin B was used to select the transformants. Conjugation between *E. coli* and the ΔphoP strain was carried out, conjugants were selected by hygromycin B, and the genomic structure of the complemented strain C-ΔphoP was confirmed by PCR analysis.

### Scanning Electron Microscopy

Spores of X47 and its derivatives were cultured on ISP2 medium, and sterile glass coverslips were inserted into the agar (Supplementary Figure S1). The coverslips were removed when the strains had grown for 48 or 72 h, and the coverslips with cultures were soaked in 2% glutaraldehyde for 2 h at room temperature. The fixed coverslips were washed three times with 0.1 M PBS buffer and treated with 1% osmic acid. Then, the coverslips were dehydrated in a critical point dryer (Quorum K850) then coated with gold and imaged with a scanning electron microscope (HITACHI Regulus 8,100).

### HPLC Analysis of the AP-3 Production

AP-3 produced by *A. pretiosum* X47 and ΔphoP strains was extracted with ethyl acetate and detected by HPLC analysis as described (Zhong et al., 2019). HPLC was performed on a Diamonsil C18 Column (250 mm × 4.6 mm) with acetonitrile-water gradient as flow phase and UV detector at 254 nm.

### RNA Extraction and RNA Sequencing

The *A. pretiosum* X47 and ΔphoP strains were cultivated on solid ISP2 medium for 60 h. To isolate RNA, the mycelium was collected and mixed with Trizol reagent. RNA extraction and DNA removal were conducted by RNA extraction kit (Vazyme). The integrity of the RNA samples was analyzed by agarose gel electrophoresis, and the sample concentrations were detected using an Agilent 2,100 bioanalyzer. RNA sequencing was conducted by Novagene Bioinformatics Technology Co., Ltd. (Beijing, China).

### Construction of a *phoP* Expression Plasmid and Purification of PhoP Protein

The coding region of the *phoP* gene of *A. pretiosum* X47 was amplified using primers PhoP-pET15bF/R, and the PCR products were inserted into pET-15b (Novagen) to generate the *phoP* expression plasmid pPhoP. Then, pPhoP was introduced into BL21 (DE3), and *the* transformants were selected using ampicillin. The expression of PhoP protein was induced by 0.5 mM isopropyl β-D-1-thiogalactopyranoside at 25°C for 3–4 h when cell density reached an OD_600_ reading of 0.4–0.6. Cell pellets were collected, and cell lysates were prepared by sonication. PhoP protein was purified on a Ni-NTA column (Sangon) and dialyzed in dialyzing buffer (50 mM NaH_2_PO_4_ and 50 mM NaCl, pH 8.0). The concentration of PhoP protein was determined with the BCA Protein Assay Kit (Beyotime).

### Electrophoretic Mobility Shift Assays

The upstream regions of *ssgB*, *flgB*, *flaD*, *cheA*, *fliM*, *swrD*, and *whiB* were amplified by 5’-biotin-labeled primer pairs (Supplementary Table S1) to generate DNA probes. For EMSAs, 100 fmol probes were incubated with PhoP protein, binding buffer, and poly(dI-dC) for 20–30 min at room temperature. The mixtures were loaded on 8% non-denaturing polyacrylamide gels, and then, DNA was transferred to nylon membranes and fixed at 120°C. After blocking and washing, the probe signals were detected by the ECL Western Blotting Analysis System (GE Life).

### Bioinformatic Analysis

The conservation of *phoP*/*phoR* genes in *A. pretiosum* X47, *S. coelicolor*, and *M. tuberculosis* H37Rv was analyzed by BLAST.^1^ PHO box-like motif sequences were predicted by MEME software (Bailey et al., 2015), and searches for PHO box-like motif in the X47 genome were conducted using PREDetector software (Hiard et al., 2007).

### Real-Time PCR

Spores of X47 were cultured on ISP2 medium with 0 and 5 mM K_2_HPO_4_ for 72 h, and then, cultures were collected. Total RNA was obtained by RNA extraction kit (Sparkeasy), and cDNA was synthesized using ReverTra Ace qPCR RT Master Mix (TOYOBO). Real-time PCR was performed by using SYBR Premix Ex Taq kit (TaKaRa) on Roche LightCycler480 thermal cycler. The amounts of cDNA were normalized to the levels of major sigma factor gene *hrdB*. Results are the means of triplet experiments.

## Results

### Identification of PhoPR in the *Actinosynnema pretiosum* X47 Strain

**Figure 1:**
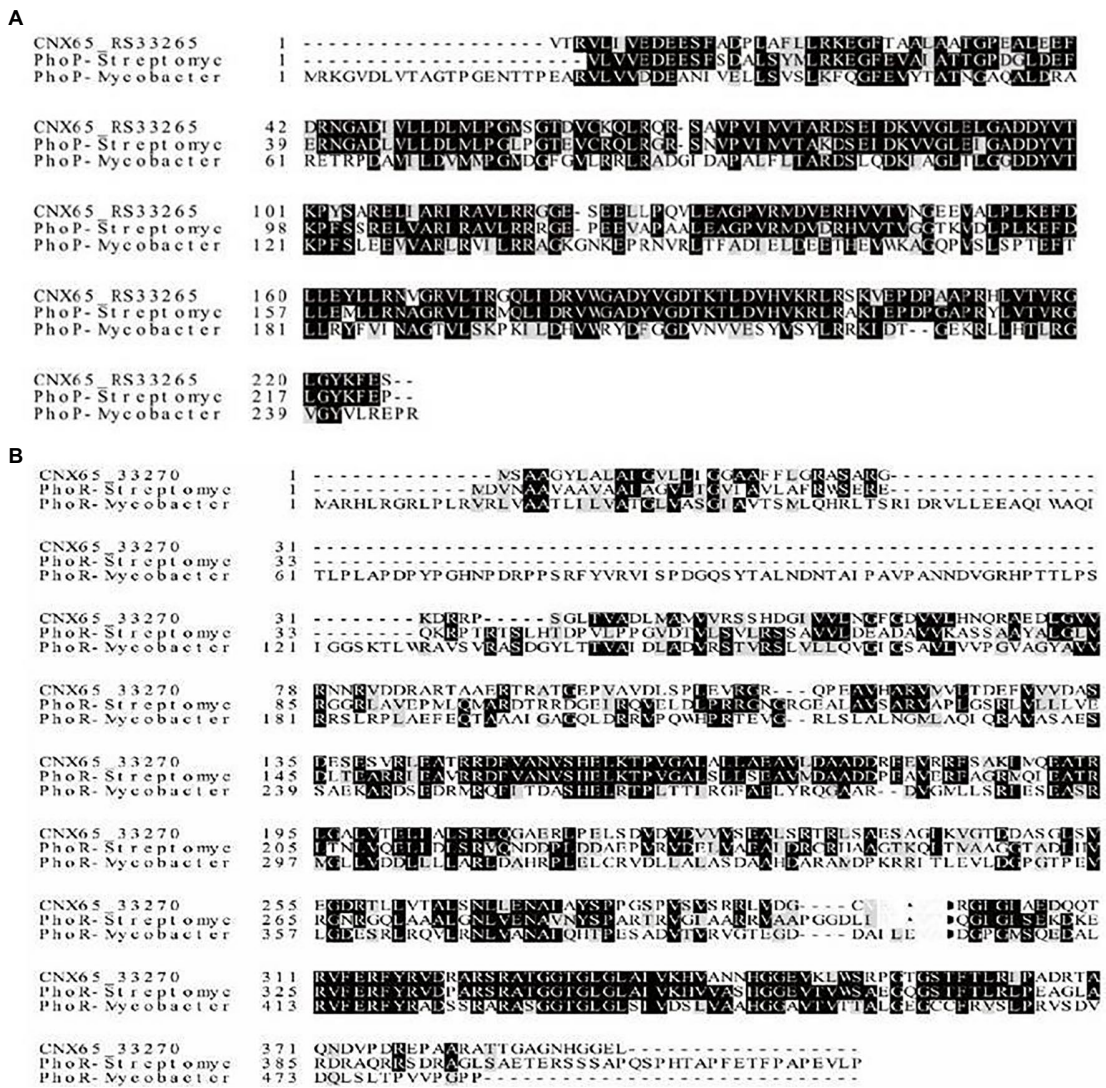
Amino acid alignment of PhoP and PhoR in *Actinosynnema pretiosum* X47. Sequence comparison of PhoP **(A)** and PhoR **(B)** of *A. pretiosum* X47 and their counterparts in *Streptomyces coelicolor* A3(2) and *Mycobacterium tuberculosis* H37Rv. Identical amino acid residues are highlighted in black, and similar residues are shown in gray.

CNX65_RS33265 and CNX65_RS33270 were identified as the *phoP* and *phoR* homologs, respectively, in the *A. pretiosum* X47 genome. Sequence alignments (Figure 1) showed that PhoP and PhoR of *A. pretiosum* X47 share 82% and 51% amino acid identity with their counterparts in *S. coelicolor* A3(2; Martin, 2004) and 73% and 35% amino acid identity with the corresponding *M. tuberculosis* H37Rv proteins (Walters et al., 2006).

### Deletion of *phoP* Accelerates the Morphological Differentiation of *Actinosynnema pretiosum*

**Figure 2:**
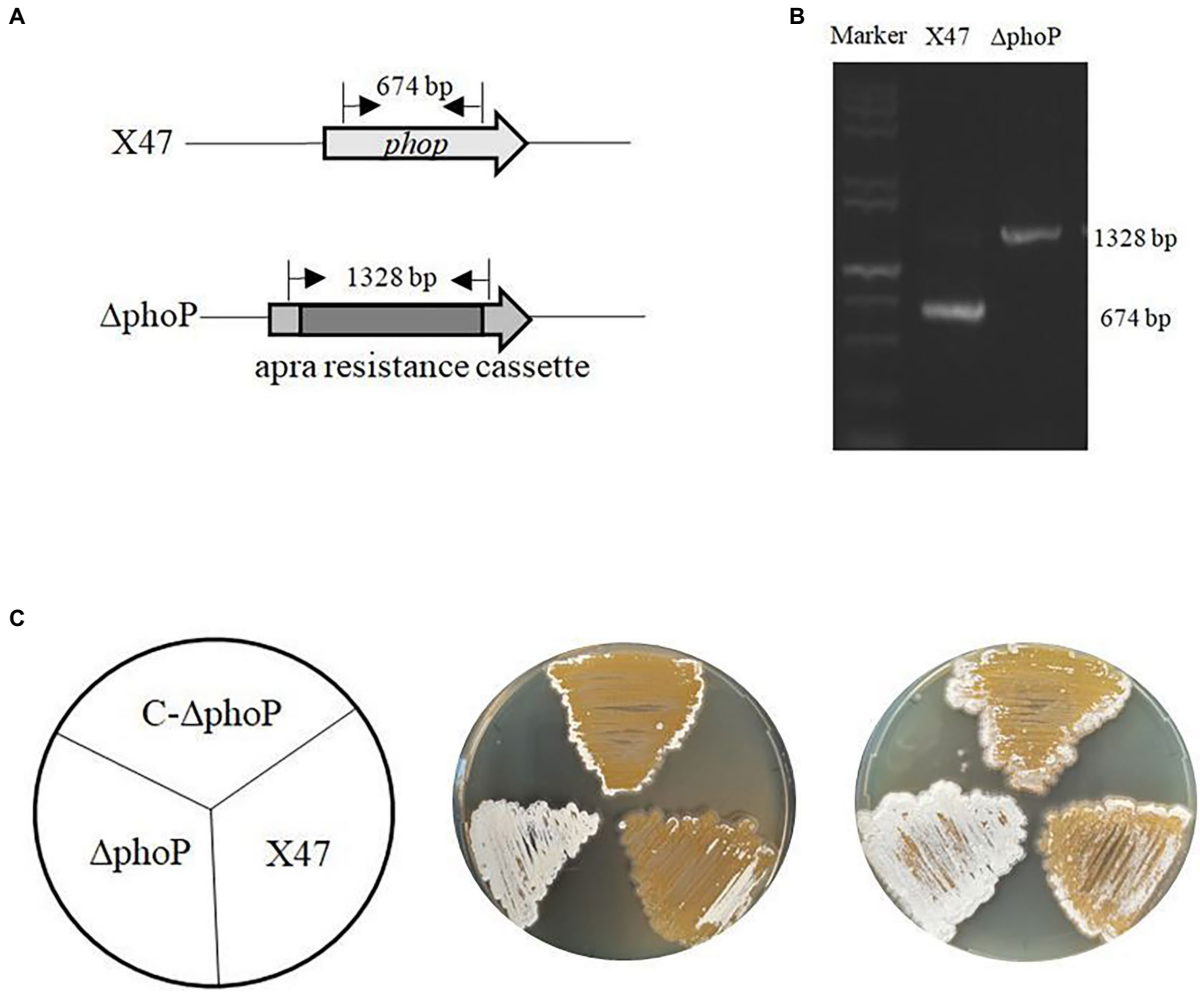
Deletion of *phoP* accelerates morphological differentiation in *A. pretiosum* X47. **(A)** Schematic design showing replacement of a 674-bp internal sequence of *phoP* with an apramycin resistance cassette. **(B)** Confirmation of the *phoP* mutation by PCR analysis with primers flanking the deleted region. PCR products were electrophoresed on an agarose gel. **(C)** Phenotypes of the wild-type *A. pretiosum* strain X47, *phoP* mutant strain ∆phoP, and complemented strain C-∆phoP grown at 30°C on ISP2 medium for 48 and 72 h.

To investigate the function of PhoP in *A. pretiosum*, a ΔphoP mutant strain, lacking part of the *phoP* coding region from position +147 to +582 downstream of translation start site, was generated, which retains *phoR* and its promoter (Figures 2A,B). Inactivation of *phoP* resulted into significant morphological differences compared to strain X47 upon growth on ISP2 solid medium (Figure 2C). The arising of aerial mycelium and production of spores occurred much earlier in the ΔphoP strain than in the wild-type strain. The ΔphoP strain produced white aerial mycelium at 48 h, which turned light yellow during spore formation at 72 h, whereas strain X47 developed mainly vegetative mycelium and had little aerial mycelium at 48 and 72 h. The *phoP*-complemented strain C-ΔphoP had a phenotype similar to that of X47 (Figure 2C). Altogether these data indicated that PhoP is a crucial regulator controlling the developmental process of *A. pretiosum*.

**Figure 3:**
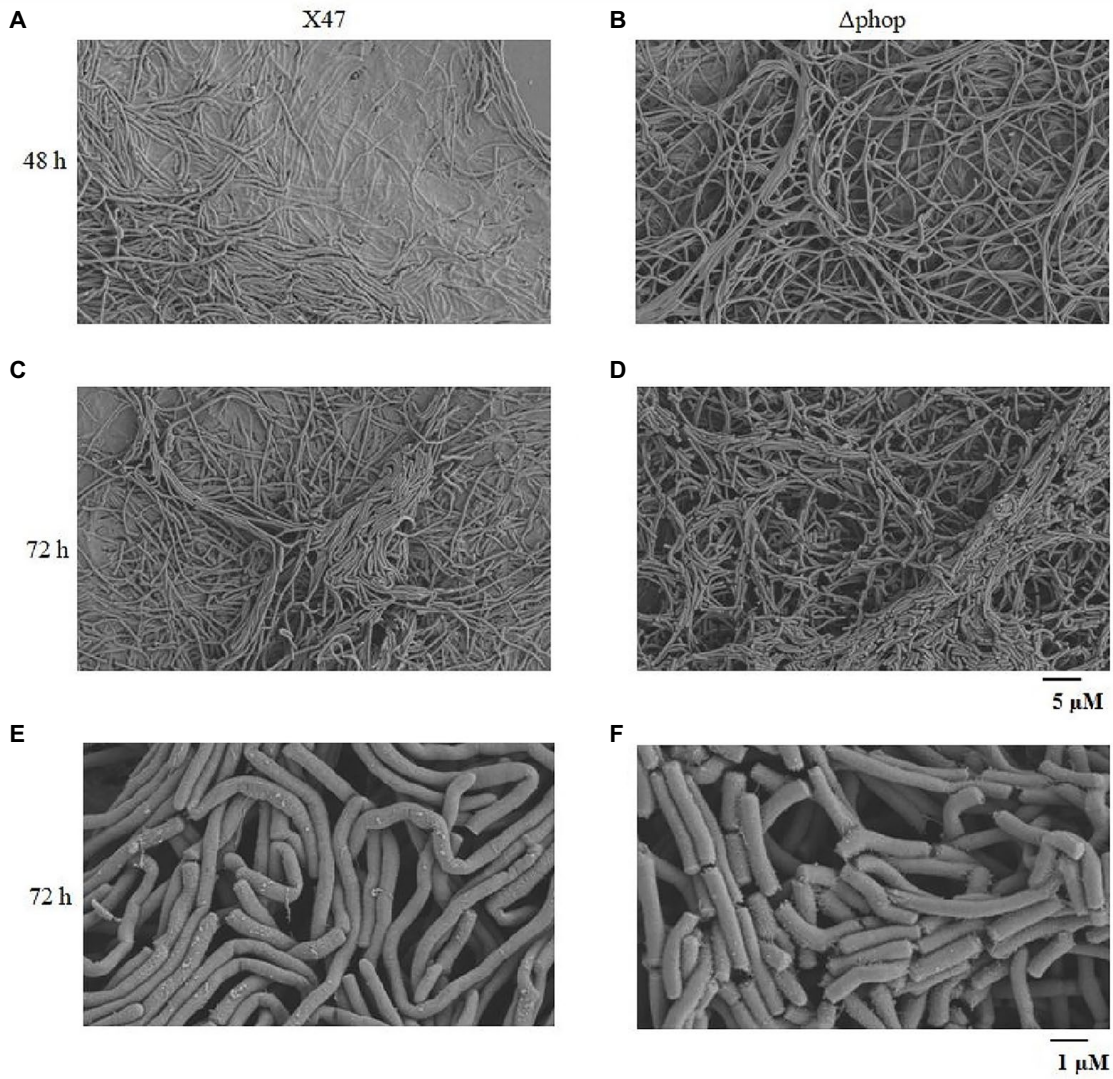
PhoP influences the formation of aerial hyphae and spores in *A. pretiosum*. **(A–D)** SEM images of strain X47 after growth on ISP2 medium for 48 h **(A)** and 72 h **(C)**, and ∆phoP strain for 48 h **(B)** and 72 h **(D)**. The scale bar is 5 μM. **(E,F)** SEM images of X47 **(E)** and ∆phoP **(F)** strains after growth on ISP2 medium for 72 h. The scale bar is 1 μM.

Scanning electron microscopy (SEM) was carried out to visualize differences in the morphological differentiation of X47 and ΔphoP grown on ISP2 medium for 48 and 72 h. The mycelium of the X47 strain mycelium grew inside the growth medium (Figure 3A), whereas the ΔphoP strain showed aerial mycelium growing into the air at 48 h (Figure 3B). By 72 h, the X47 strain had produced only a small amount of aerial mycelium, whereas ΔphoP strain exhibited much more aerial mycelium (Figures 3C,D). While as the resolution images of samples cultured by 72 h were enhanced, it was found that the aerial mycelium of ΔphoP has separated into spores, and no spore formation was observed in X47 strain (Figures 3E,F). These results indicated that the formation of aerial mycelium and spores was accelerated in the ΔphoP mutant of *A. pretiosum*.

The productions of AP-3 in X47 and ΔphoP strains were analyzed by HPLC. The results (Supplementary Figure S2) showed that there is no significant difference in yield of AP-3 between X47 and ΔphoP strains, suggesting that PhoP is not essential for the AP-3 production under tested conditions.

### *phoP* Deletion Alters Global Transcriptional Expression

To investigate the mechanism of morphological differentiation caused by PhoP, the transcription patterns of the ∆phoP and X47 strains grown on ISP2 medium for 60 h were compared by RNA-seq analysis. Compare to the X47 strain, 222 genes were differentially expressed in ∆phoP. Among these genes, 122 genes were downregulated, and 100 genes were upregulated. The expression of gene clusters responsible for the structure, assembly, and motility of flagella was remarkably higher in ∆phoP, including *flgBCDGKLN*, *flaD*, *fliD-R*, *motA*, and *swrD* (Table 1). In addition, *whiB* and *ssgB*, which are involved in cell division and sporulation of *Streptomyces*, and the chemotaxis-related genes *cheA*, *cheW*, *cheX*, and *cheY* were significantly upregulated in the ∆phoP strain. Overall, the results of RNA-seq indicated that PhoP negatively regulates the transcription of genes required for chemotaxis and for the formation of flagella and spores.

**Table 1:** PhoP deletion alters the expression of developmental and chemotaxis-related genes in *A. pretiosum*.

Gene ID	Gene	Function	Fold change (ΔphoP/X47)	Q-value
CNX65_RS10075	ssgB^*^	SsgA family sporulation/cell division regulator	3.60	<0.001
CNX65_RS11775	*flhA*	FHIPEP family type III secretion protein	7.78	<0.001
CNX65_RS11785	*cheY*	Response regulator	16.62	<0.001
CNX65_RS11790	*cheX*	Chemotaxis protein CheX	15.52	<0.001
CNX65_RS11795	*cheY*	Response regulator	13.40	<0.001
CNX65_RS11800	*cheR*	Protein-glutamate O-methyltransferase CheR	11.04	<0.001
CNX65_RS11805	*cheB*	Chemotaxis response regulator protein-glutamate methylesterase	8.82	<0.001
CNX65_RS11815	*mcpQ*	Methyl-accepting chemotaxis protein	9.46	<0.001
CNX65_RS11820	*cheW*	Chemotaxis protein CheW	11.10	<0.001
CNX65_RS11825	*cheA*	Chemotaxis protein CheA/chemotaxis protein CheW	9.84	<0.001
CNX65_RS11855	*flhB*	EscU/YscU/HrcU family type III secretion system export apparatus switch protein	6.43	<0.001
CNX65_RS11860	*fliR*	Flagellar biosynthetic protein FliR	5.96	<0.001
CNX65_RS11865	*fliQ*	Flagellar biosynthesis protein FliQ	6.20	<0.001
CNX65_RS11870	*fliP*	Flagellar type III secretion system pore protein FliP	5.84	<0.001
CNX65_RS11875	*fliO*	FliO/MopB family protein	5.97	<0.001
CNX65_RS11880	*fliN*	Flagellar motor switch protein FliN	5.24	<0.001
CNX65_RS11885	*fliM*	Flagellar motor switch protein FliM	4.84	<0.001
CNX65_RS11890	*fliL*	Flagellar basal body-associated FliL family protein	18.90	<0.001
CNX65_RS11895	*ompA*	OmpA family protein	16.37	<0.001
CNX65_RS11900	*motA*	Motility protein A	16.13	<0.001
CNX65_RS11905	*flbD*/*swrD*	Flagellar FlbD family protein	13.73	<0.001
CNX65_RS11910	*flgG*	Flagellar basal-body rod protein FlgG	16.40	<0.001
CNX65_RS11915	*flgD*	Flagellar hook capping protein	15.07	<0.001
CNX65_RS11920	*fliK*	Flagellar hook-length control protein FliK	10.93	<0.001
CNX65_RS11925	*nlpC*	Transglycosylase SLT domain-containing protein	9.81	<0.001
CNX65_RS11930	*tolA*	Cell envelope biogenesis protein TolA	8.57	<0.001
CNX65_RS11935	*fliI*	FliI/YscN family ATPase	8.52	<0.001
CNX65_RS11940	*fliH*	Flagellar assembly protein	11.69	<0.001
CNX65_RS11945	*fliG*	Flagellar motor switch protein FliG	11.69	<0.001
CNX65_RS11950	*fliF*	Flagellar M-ring protein FliF	8.72	<0.001
CNX65_RS11955	*fliE*	Flagellar hook-basal-body complex protein FliE	7.07	<0.001
CNX65_RS11960	*flgC*	Flagellar basal-body rod protein FlgC	8.09	<0.001
CNX65_RS11965	flgB^*^	Flagellar basal-body rod protein FlgB	8.07	<0.001
CNX65_RS11975	*fliS*	Flagellar export chaperone FliS	9.06	<0.001
CNX65_RS11980	*fliD*	Flagellar filament capping protein FliD	9.76	<0.001
CNX65_RS11985	flaD^*^	Flagellin	15.58	<0.001
CNX65_RS11990	*fliA*	Sigma-70 family RNA polymerase sigma factor	12.82	<0.001
CNX65_RS11995	*flgN*	Flagellar export chaperone FlgN	8.94	<0.001
CNX65_RS12000	*flgK*	Flagellar hook-associated protein FlgK	8.62	<0.001
CNX65_RS12005	*flgL*	Flagellar hook-associated protein 3	5.93	<0.001
CNX65_RS12010	*fliW*	Flagellar assembly protein FliW	4.37	<0.001
CNX65_RS12015	*csrA*	Carbon storage regulator CsrA	5.57	<0.001
CNX65_RS32100	*whiB*	WhiB family transcriptional regulator	5.62	<0.001

* One or more putative PHO boxes in the promoter.

### PhoP Binds to the Promoters of *flgB*, *flaD*, and *ssgB*

**Figure 4:**
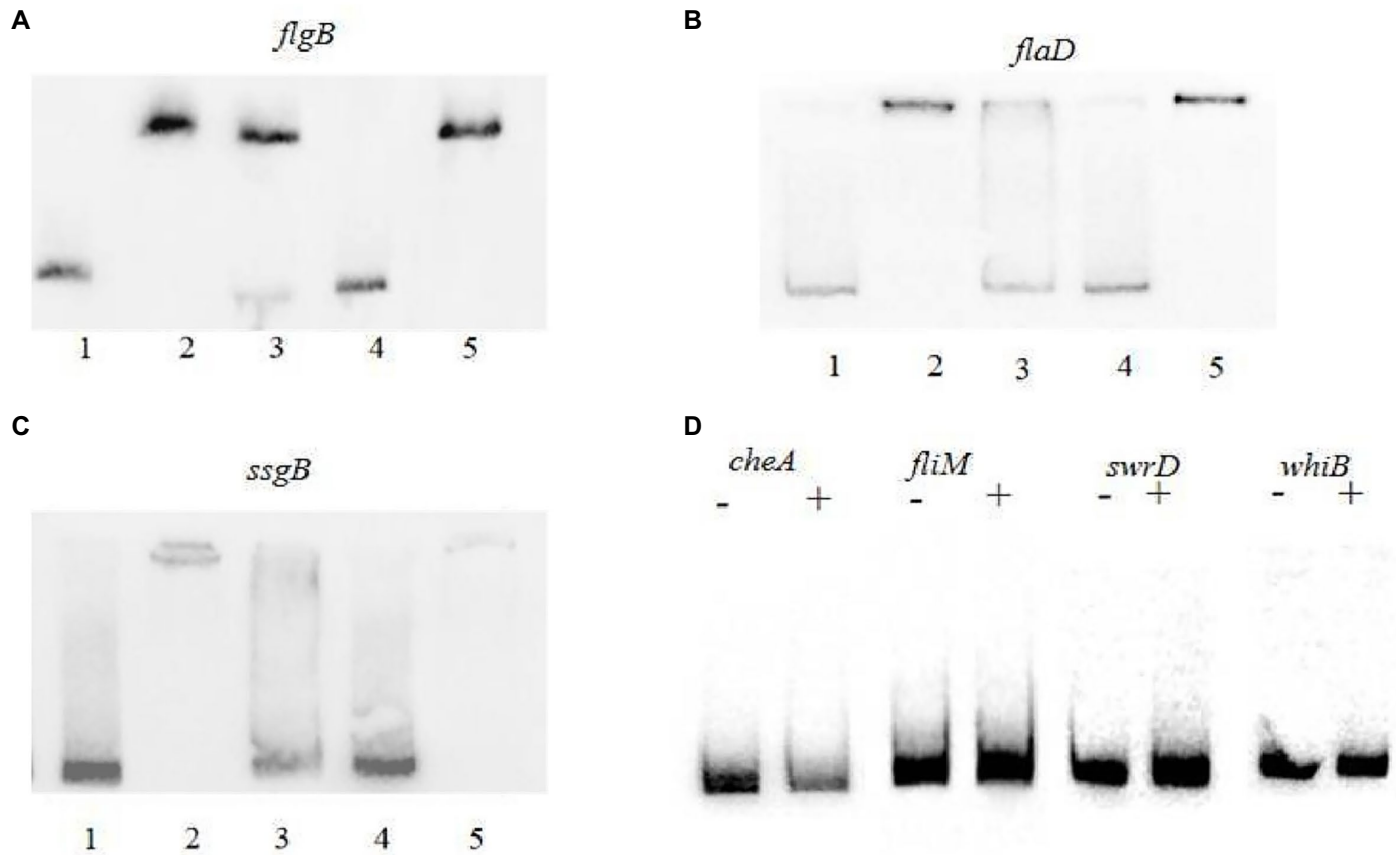
EMSAs with PhoP protein of *A. pretiosum* X47. **(A–C)** Binding of PhoP with the upstream sequences of *flgB* **(A)**, *flaD* **(B)**, and *ssgB* **(C)**. The probes were incubated with no protein (lane 1), 1.0 μg protein (lane 2), 1.0 μg protein (lanes 3–5), 50-fold excess of unlabeled specific probe (lane 3), 200-fold excess of unlabeled specific probe (lane 4), and 200-fold excess of unlabeled unspecific probe (lane 5). **(D)** Binding of PhoP with the upstream sequences of *cheA*, *fliM*, *swrD*, and *whiB*. The probes were incubated with no protein (−) or 4 μg protein (+).

The above analysis demonstrated that PhoP controls multiple genes related to morphological development. Based on the transcriptional changes (Table 1) and gene arrangements (Supplementary Figure S3), it was deduced that flagellum biosynthesis and motility genes were contained within several operons (*cheAW*, *swrD-motA-ompA-fliL*, *fliMOPQR-flhB*, *flaD-fliD-fliS*, and *flgBC-fliEFGHI-tolA-nlpC-fliK-flgD-flgG*). To determine whether PhoP directly regulates the expression of these genes, electrophoretic mobility shift assays (EMSAs) were performed. His_6_-PhoP protein was purified, and 200-bp sequences upstream of the *flgB*, *flaD*, *ssgB*, *cheA*, *fliM*, *swrD*, and *whiB* genes were amplified and labeled with biotin to create the probes. EMSA results (Figures 4A–C) showed that the *flgB*, *flaD*, and *ssgB* probes were shifted when incubated with 1.0 μg His_6_-PhoP. As a control, for each target gene, excess unlabeled specific DNA fragments were added to the reactions and resulted into the apparition of free un-shifted probe demonstrating that the binding was specific, while excess unlabeled unspecific DNA fragments in the reaction did not influence the bindings. The above results suggested that PhoP specifically binds to the promoters of *flgB*, *flaD*, and *ssgB*. However, no shift was observed when PhoP was incubated with the promoters of *cheA*, *fliM*, *whiB*, or *swrD* (Figure 4D), suggesting that PhoP controls the transcription of these genes indirectly.

### PHO Box-Like Motifs Are Present in the Promoters of *flgB*, *flgN*, and *ssgB*

**Figure 5:**
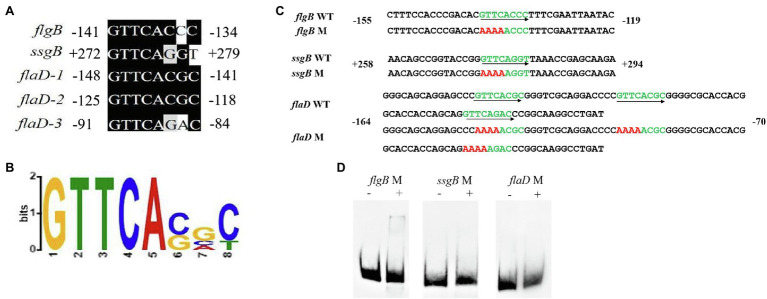
Consensus binding sequence for PhoP in *A. pretiosum* and mutational analysis of predicted PHO box-like motif in the promoters of *flgB*, *ssgB*, and *flaD*. **(A)** Alignment of putative PHO boxes box-like motif in the promoters of *flgB*, *ssgB*, and *flaD*. Conserved nucleotides are indicated by a dark background. **(B)** Consensus binding sequence for PhoP comprising eight nucleotides, based on the alignment in panel **(A)**. **(C)** Mutation analyses of putative PHO box-like motif in the promoters of *flgB*, *ssgB*, and *flaD*. Putative PHO box-like motifs are shown in green and mutagenized nucleotides are in red. The orientation of consensus sequences is indicated by arrow. **(D)** EMSAs with mutant probes. The probes were incubated with no protein (−) or 4 μg protein (+).

PhoP controls the transcription of target genes by binding to the PHO box, a conserved motif found in their promoters (Santos-Beneit, 2015). The results of sequence analysis indicated that the putative PHO box-like motifs identified in *Streptomyces* also existed in the promoter regions of *flgB*, *flaD*, and *ssgB* genes in *A. pretiosum*. In the *flgB* promoter, the putative PHO box-like motif (GTTCACCC) was located from position −141 to −134 bp upstream from transcription start position (TSP; Figure 5A), and for the *ssgB* promoter, the putative PHO box-like motif (GTTCAGGT) was located from position 272 to 279 bp downstream from TSP. Three PHO box-like motifs (GTTCACGC, GTTCACGC, and GTTCAGAC) were identified in the promoter of *flaD*, with the last one ending −84 bp upstream from TSP (Figure 5A). While eight consensus binding sequences with five conserved nucleotides for PhoP were revealed (Figure 5B). To confirm the role of PHO box-like motifs identified in this study in PhoP binding, EMSAs were performed by using probes containing mutation in the promoters of *flgB*, *flaD*, and *ssgB* genes. Mutation of four conserved nucleotides in the putative PHO box-like motifs severely reduced PhoP binding (Figures 5C,D), suggesting that the motifs are essential for PhoP binding to the promoters. These PHO box-like motifs have the 8-bp consensus motif of GTTCACNC, and hundreds of sites with this motif were identified in intergenic regions in the genome of strain X47 using PREDetector software (Hiard et al., 2007). Supplementary Table S2 shows a partial list of PHO box-like motifs in *A. pretiosum*, and the range of target genes further indicates that PhoP is a global regulator.

### The Transcription of *phoP*/*phoR* Is Induced Under Pi Limitation

**Figure 6:**
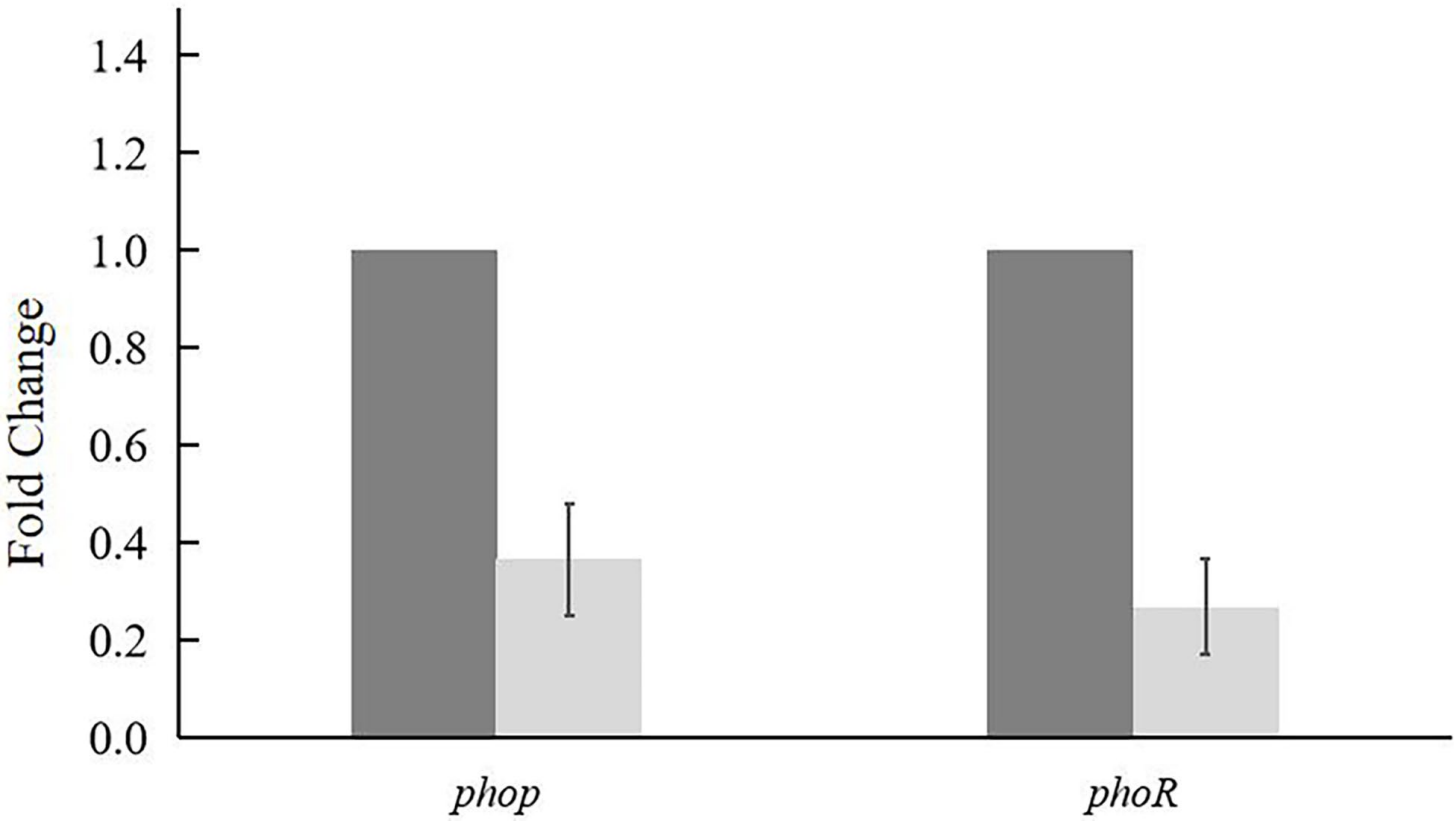
Transcriptional analysis of *phop* and *phoR* in X47 strain was analyzed by real-time PCR. X47 strain was cultured on ISP2 with 0 mM (dark gray bars) and 5 mM (light gray bars) K_2_HPO_4_ for 72 h. Expression of major sigma factor gene *hrdB* was used as an internal control. Results are the means of triplet experiments.

The concentration of Pi in ISP2 liquid medium was determined, which is 0.44 ± 0.05 μM, suggesting that Pi in ISP2 medium is scarce. Real-time PCR assays (Figure 6) showed that the expression of *phop*/*phoR* in X47 strain was significantly decreased when 5 mM K_2_HPO_4_ was added in the medium, which indicated that the transcription of *phop*/*phoR* was upregulated under the condition of Pi limitation.

## Discussion

PhoPR is known to influence the growth of *Streptomyces*; for example, deletion of *phoP* or *phoPR* led to poor growth and fewer spores in *S. avermitilis* as well as in *S. lividans* (Smirnov et al., 2015; Yang et al., 2015). In this study, we demonstrated that deletion of phoP accelerated morphological differentiation of *A. pretiosum* strain X47, another member of the actinobacteria genera, indicating that PhoP plays an essential role in the regulation of the developmental process of this species. Several genes involved in the differentiation process of various *Streptomyces* species, such as *bldA*, *bldC*, *bldD*, *bldM*, and *whiH*, were shown to be negatively regulated by PhoP (Flardh and Buttner, 2009; Martin et al., 2012). Although the morphological development of *A. pretiosum* is similar to that of *Streptomyces*, no homologs of *bldC*, *bldD*, or *bldM* were found in *A. pretiosum*, suggesting that PhoP regulates the growth of this species in a different way. However, homologs of the genes *ssgB* and *whiB*, which are related to the cellular differentiation of *Streptomyces*, were identified in *A. pretiosum* (Davis and Chater, 1992; Kormanec et al., 1998; Molle et al., 2000; Keijser et al., 2003; Sevcikova and Kormanec, 2003), and we found that these *ssgB* and *whiB* homologs were upregulated in the ∆phoP mutant of *A. pretiosum* and that PhoP directly binds the *ssgB* promoter, suggesting that PhoP could affect development of *A. pretiosum* by controlling the transcription of these two genes.

Furthermore, PhoP, a response regulator from TCS PhoPQ, was previously shown to control positively the transcription of genes involved in chemotaxis (*cheW*, *cheA*, and *cheY*) and flagellum formation (*flgBCDEFGHIJKL*) in *Xanthomonas citri* (Wei et al., 2019). In *E. coli* APEC, the transcription of genes involved in flagellar assembly (*motA*/*flgN*/*fliA*/*flgM*) and *csgA* gene, encoding a fimbrial protein was downregulated in *phoP* deletion strain, resulting in reduced the formation of biofilm (Yin et al., 2019). Flagellum- and chemotaxis-related genes were identified in the genome of the *A. pretiosum* X47 strain (GenBank Accession number CP023445; Zhong et al., 2019). RNA-seq indicated that transcription of most flagellum-related and chemotaxis-related genes was upregulated in the *phoP* deletion mutant (Table 1). Interestingly, whereas PhoP regulates positively these groups of genes in *X. citri subsp. citri* and APEC (Wei et al., 2019; Yin et al., 2019), it regulates negatively their expression in *A. pretiosum*. It is deduced that the different effects of PhoP in *A. pretiosum*, *X. citri*, and APEC on chemiotaxis and flagella formation genes could be related to the different strains or different cultured conditions.

The DNA-binding sites of PhoP, named PHO boxes, have been well characterized, with variation detected in their consensus sequences among different species. In *M. tuberculosis*, PhoP specifically binds to a consensus sequence with a 7-bp direct repeat separated by a 4-bp spacer, TCACAGC (n4) TCACAGC (Gonzalo-Asensio et al., 2008; Gupta et al., 2009; He and Wang, 2014). The conserved sequence for PhoP-binding sites in *E. coli* is TGTTTA (n5) TGTTTA (Kato et al., 1999; Zhou et al., 2003), and in *S. coelicolor*, the PHO box consists of 11-bp direct repeats, with the sequence GTTCACC comprising the most conserved motif of the repeats (Sola-Landa et al., 2005, 2008; Santos-Beneit et al., 2008). Our data showed that PhoP of the *A. pretiosum* X47 strain directly binds to the promoters of the *flgB*, *flaD*, and *ssgB* genes, and these promoters contained PHO box-like motifs with an 8-bp consensus sequence of GTTCACGC, which is similar to the conserved sequences of the PHO boxes in *S. coelicolor*.

In this study, we confirmed that PhoPR is involved in regulating the growth and development of the *A. pretiosum* X47. According to the sequence alignment and maximum likelihood evolution analysis (Supplementary Figure S4), PhoR in *A. pretiosum* is relative conserved in *Pseudonocardia*, and it has high sequence similarity with its counterparts in *Streptomyces* (nearly 50% identity), *Bacillus subtilis* (34.58% identity), and *E. coli* (36.42% identity), which sense Pi concentration in the medium (Martin et al., 2017; Devine, 2018; Gardner and McCleary, 2019). The similarities are significantly higher than that of PhoQ in *E. coli*, *X. citri subsp. citri*, *Salmonella*, and other strains, although TCS PhoPQ was found to involve in regulating flagella formation and motility (Tu et al., 2016; Wei et al., 2019; Yin et al., 2019). The evolution relationship of PhoR in *A. pretiosum* is closer to that of *S. coelicolor*, and the conserved binding sites of PhoP in *A. pretiosum* is similar to that of *S. coelicolor*, suggesting that the TCS PhoPR in *A. pretiosum* is probably to sense Pi concentration which is similar to that in *S. coelicolor*. The Pi in ISP2 medium used in this study is insufficient and the transcription of *phoP* was induced under Pi limitation, indicating that Pi limitation could be the signal that PhoPR senses in X47 strain.

A genome-wide search of *A. pretiosum* X47 revealed many other intergenic regions containing PHO box-like motifs (Supplementary Table S2), and many of the genes with these upstream PHO box-like motifs are known to be targets of PhoP in other species, suggesting that PhoP is also a global regulator in *A. pretiosum*. For example, PHO box-like motifs were identified in the upstream sequences of *glnA*, *phoU*, and *pstS* in the X47 strain genome. In *S. coelicolor*, PhoP directly regulates the transcription of *glnA*, *phoU*, and *pstS* (Apel et al., 2007; Rodriguez-Garcia et al., 2009; Sola-Landa et al., 2013). *glnA* encodes a glutamine synthetase type I involved in nitrogen metabolism and is negatively regulated by PhoP, whereas PhoU (phosphate transport regulator) and PstS (secreted phosphate-binding protein) that are involved into phosphorus metabolism, are positively regulated by PhoP. *glnA*, *phoU*, and *pstS* may also be target genes of PhoP in *A. pretiosum*, although the transcription levels of these genes were not influenced under the conditions used in this study. However, further studies are needed to fully understand the functions of PhoPR in *A. pretiosum* and how this TCS and its target genes enable this bacterium to respond to various environmental conditions.

## Data Availability Statement

The datasets presented in this study can be found in online repositories. The names of the repository/repositories and accession number(s) can be found in the article/Supplementary Material.

## Author Contributions

GC contributed to conception and design of the study. PZ prepared the manuscript. KZ performed the experiment. YL and XM analyzed the data. GZ and JF revised the article. All authors contributed to the article and approved the submitted version.

## Funding

This work was supported by the Shandong Provincial Natural Science Foundation (no. ZR2021QC109) and the Academic Promotion Programme of Shandong First Medical University (no. LJ001).

## Conflict of Interest

The authors declare that the research was conducted in the absence of any commercial or financial relationships that could be construed as a potential conflict of interest.

## Publisher’s Note

All claims expressed in this article are solely those of the authors and do not necessarily represent those of their affiliated organizations, or those of the publisher, the editors and the reviewers. Any product that may be evaluated in this article, or claim that may be made by its manufacturer, is not guaranteed or endorsed by the publisher.

## Footnotes

^1^ https://blast.ncbi.nlm.nih.gov/Blast.cgi

## References

[ref1] Apel A. K. Sola-Landa A. Rodriguez-Garcia A. Martin J. F. (2007). Phosphate control of phoA, phoC and phoD gene expression in *Streptomyces coelicolor* reveals significant differences in binding of PhoP to their promoter regions. Microbiology 153, 3527–3537. doi: 10.1099/mic.0.2007/007070-0, PMID: 17906150

[ref2] Bailey T. L. Johnson J. Grant C. E. Noble W. S. (2015). The MEME suite. Nucleic Acids Res. 43, W39–W49. doi: 10.1093/nar/gkv416, PMID: 25953851 PMC4489269

[ref3] Barreales E. G. Payero T. D. de Pedro A. Aparicio J. F. (2018). Phosphate effect on filipin production and morphological differentiation in *Streptomyces filipinensis* and the role of the PhoP transcription factor. PLoS One 13:e208278. doi: 10.1371/journal.pone.0208278, PMID: 30521601 PMC6283541

[ref4] Bierman M. Logan R. O'Brien K. Seno E. T. Rao R. N. Schoner B. E. (1992). Plasmid cloning vectors for the conjugal transfer of DNA from *Escherichia coli* to *Streptomyces* spp. Gene 116, 43–49. doi: 10.1016/0378-1119(92)90627-2, PMID: 1628843

[ref5] Broset E. Martin C. Gonzalo-Asensio J. (2015). Evolutionary landscape of the *Mycobacterium tuberculosis* complex from the viewpoint of PhoPR: implications for virulence regulation and application to vaccine development. MBio 6, e01289–e01315. doi: 10.1128/mBio.01289-15, PMID: 26489860 PMC4620462

[ref6] Davis N. K. Chater K. F. (1992). The *Streptomyces coelicolor* whiB gene encodes a small transcription factor-like protein dispensable for growth but essential for sporulation. Mol. Gen. Genet. 232, 351–358. doi: 10.1007/BF00266237, PMID: 1316997

[ref7] den Hengst C. D. Tran N. T. Bibb M. J. Chandra G. Leskiw B. K. Buttner M. J. (2010). Genes essential for morphological development and antibiotic production in *Streptomyces coelicolor* are targets of BldD during vegetative growth. Mol. Microbiol. 78, 361–379. doi: 10.1111/j.1365-2958.2010.07338.x, PMID: 20979333

[ref8] Devine K. M. (2018). Activation of the PhoPR-mediated response to phosphate limitation is regulated by wall teichoic acid metabolism in *Bacillus subtilis*. Front. Microbiol. 9:2678. doi: 10.3389/fmicb.2018.02678, PMID: 30459743 PMC6232261

[ref9] Flardh K. Buttner M. J. (2009). Streptomyces morphogenetics: dissecting differentiation in a filamentous bacterium. Nat. Rev. Microbiol. 7, 36–49. doi: 10.1038/nrmicro1968, PMID: 19079351

[ref10] Galperin M. Y. (2010). Diversity of structure and function of response regulator output domains. Curr. Opin. Microbiol. 13, 150–159. doi: 10.1016/j.mib.2010.01.005, PMID: 20226724 PMC3086695

[ref11] Gardner S. G. McCleary W. R. (2019). Control of the phoBR Regulon in *Escherichia coli*. EcoSal Plus 8, 1–20. doi: 10.1128/ecosalplus.ESP-0006-2019 PMC11573284 31520469

[ref12] Gonzalo-Asensio J. Soto C. Y. Arbues A. Sancho J. Del C. M. M. Garcia M. J.. (2008). The *Mycobacterium tuberculosis* phoPR operon is positively autoregulated in the virulent strain H37Rv. J. Bacteriol. 190, 7068–7078. doi: 10.1128/JB.00712-08, PMID: 18757548 PMC2580713

[ref13] Gupta S. Pathak A. Sinha A. Sarkar D. (2009). *Mycobacterium tuberculosis* PhoP recognizes two adjacent direct-repeat sequences to form head-to-head dimers. J. Bacteriol. 191, 7466–7476. doi: 10.1128/JB.00669-09, PMID: 19820095 PMC2786591

[ref14] Hackl S. Bechthold A. (2015). The gene bldA, a regulator of morphological differentiation and antibiotic production in streptomyces. Arch. Pharm. 348, 455–462. doi: 10.1002/ardp.201500073, PMID: 25917027

[ref15] Hasegawa T. T. S. H. (1983). Motile actinomycetes: *Actinosynnema pretiosum* sp. nov., subsp. nov., and *Actinosynnema pretiosum* subsp. pretiosum subsp. auranticum subsp. nov. Int. J. Syst. Bacteriol. 2, 314–320.

[ref16] He X. Wang S. (2014). DNA consensus sequence motif for binding response regulator PhoP, a virulence regulator of *Mycobacterium tuberculosis*. Biochemistry 53, 8008–8020. doi: 10.1021/bi501019u, PMID: 25434965 PMC4283936

[ref17] He Y. Wang Z. Bai L. Liang J. Zhou X. Deng Z. (2010). Two pHZ1358-derivative vectors for efficient gene knockout in streptomyces. J. Microbiol. Biotechnol. 20, 678–682. doi: 10.4014/jmb.0910.10031, PMID: 20467238

[ref18] Hiard S. Maree R. Colson S. Hoskisson P. A. Titgemeyer F. van Wezel G. P.. (2007). PREDetector: a new tool to identify regulatory elements in bacterial genomes. Biochem. Biophys. Res. Commun. 357, 861–864. doi: 10.1016/j.bbrc.2007.03.180, PMID: 17451648

[ref19] Kato A. Tanabe H. Utsumi R. (1999). Molecular characterization of the PhoP-PhoQ two-component system in *Escherichia coli* K-12: identification of extracellular Mg^2+^-responsive promoters. J. Bacteriol. 181, 5516–5520. doi: 10.1128/JB.181.17.5516-5520.1999, PMID: 10464230 PMC94065

[ref20] Keijser B. J. Noens E. E. Kraal B. Koerten H. K. van Wezel G. P. (2003). The *Streptomyces coelicolor* ssgB gene is required for early stages of sporulation. FEMS Microbiol. Lett. 225, 59–67. doi: 10.1016/S0378-1097(03)00481-6, PMID: 12900022

[ref21] Kieser T. B. M. J. (2000). Practical Streptomyces Genetics. Norwich: John Innes Foundation.

[ref22] Kormanec J. Sevcikova B. Sprusansky O. Benada O. Kofronova O. Novakova R.. (1998). The *Streptomyces aureofaciens* homologue of the whiB gene is essential for sporulation; its expression correlates with the developmental stage. Folia Microbiol. 43, 605–612. doi: 10.1007/BF02816376, PMID: 10069009

[ref23] Li S. Lu C. Chang X. Shen Y. (2016). Constitutive overexpression of asm18 increases the production and diversity of maytansinoids in *Actinosynnema pretiosum*. Appl. Microbiol. Biotechnol. 100, 2641–2649. doi: 10.1007/s00253-015-7127-7, PMID: 26572523

[ref24] Liu T. Jin Z. Wang Z. Chen J. Wei L. J. Hua Q. (2020). Metabolomics analysis of *Actinosynnema pretiosum* with improved AP-3 production by enhancing UDP-glucose biosynthesis. J. Biosci. Bioeng. 130, 36–47. doi: 10.1016/j.jbiosc.2020.02.013, PMID: 32179024

[ref25] Ma J. Zhao P. J. Shen Y. M. (2007). New amide N-glycosides of ansamitocins identified from *Actinosynnema pretiosum*. Arch. Pharm. Res. 30, 670–673. doi: 10.1007/BF02977625, PMID: 17679541

[ref26] Martin J. F. (2004). Phosphate control of the biosynthesis of antibiotics and other secondary metabolites is mediated by the PhoR-PhoP system: an unfinished story. J. Bacteriol. 186, 5197–5201. doi: 10.1128/JB.186.16.5197-5201.2004, PMID: 15292120 PMC490900

[ref27] Martin K. Muller P. Schreiner J. Prince S. S. Lardinois D. Heinzelmann-Schwarz V. A.. (2014). The microtubule-depolymerizing agent ansamitocin P3 programs dendritic cells toward enhanced anti-tumor immunity. Cancer Immunol. Immunother. 63, 925–938. doi: 10.1007/s00262-014-1565-4, PMID: 24906866 PMC11029065

[ref28] Martin J. F. Rodriguez-Garcia A. Liras P. (2017). The master regulator PhoP coordinates phosphate and nitrogen metabolism, respiration, cell differentiation and antibiotic biosynthesis: comparison in *Streptomyces coelicolor* and *Streptomyces avermitilis*. J. Antibiot. 70, 534–541. doi: 10.1038/ja.2017.19, PMID: 28293039

[ref29] Martin J. F. Santos-Beneit F. Rodriguez-Garcia A. Sola-Landa A. Smith M. C. Ellingsen T. E.. (2012). Transcriptomic studies of phosphate control of primary and secondary metabolism in *Streptomyces coelicolor*. Appl. Microbiol. Biotechnol. 95, 61–75. doi: 10.1007/s00253-012-4129-6, PMID: 22622839

[ref30] Molle V. Palframan W. J. Findlay K. C. Buttner M. J. (2000). WhiD and WhiB, homologous proteins required for different stages of sporulation in *Streptomyces coelicolor* A3. J. Bacteriol. 182, 1286–1295. doi: 10.1128/JB.182.5.1286-1295.2000, PMID: 10671449 PMC94414

[ref31] Perez E. Samper S. Bordas Y. Guilhot C. Gicquel B. Martin C. (2001). An essential role for phoP in *Mycobacterium tuberculosis* virulence. Mol. Microbiol. 41, 179–187. doi: 10.1046/j.1365-2958.2001.02500.x, PMID: 11454210

[ref32] Rodriguez-Garcia A. Barreiro C. Santos-Beneit F. Sola-Landa A. Martin J. F. (2007). Genome-wide transcriptomic and proteomic analysis of the primary response to phosphate limitation in *Streptomyces coelicolor* M145 and in a DeltaphoP mutant. Proteomics 7, 2410–2429. doi: 10.1002/pmic.200600883, PMID: 17623301

[ref33] Rodriguez-Garcia A. Sola-Landa A. Apel K. Santos-Beneit F. Martin J. F. (2009). Phosphate control over nitrogen metabolism in *Streptomyces coelicolor*: direct and indirect negative control of glnR, glnA, glnII and amtB expression by the response regulator PhoP. Nucleic Acids Res. 37, 3230–3242. doi: 10.1093/nar/gkp162, PMID: 19321498 PMC2691820

[ref34] Ryding N. J. Kelemen G. H. Whatling C. A. Flardh K. Buttner M. J. Chater K. F. (1998). A developmentally regulated gene encoding a repressor-like protein is essential for sporulation in *Streptomyces coelicolor* A3(2). Mol. Microbiol. 29, 343–357. doi: 10.1046/j.1365-2958.1998.00939.x, PMID: 9701826

[ref35] Ryndak M. Wang S. Smith I. (2008). PhoP, a key player in *Mycobacterium tuberculosis* virulence. Trends Microbiol. 16, 528–534. doi: 10.1016/j.tim.2008.08.006, PMID: 18835713

[ref36] Santos-Beneit F. (2015). The pho regulon: a huge regulatory network in bacteria. Front. Microbiol. 6:402. doi: 10.3389/fmicb.2015.00402, PMID: 25983732 PMC4415409

[ref37] Santos-Beneit F. Rodriguez-Garcia A. Franco-Dominguez E. Martin J. F. (2008). Phosphate-dependent regulation of the low- and high-affinity transport systems in the model actinomycete *Streptomyces coelicolor*. Microbiology 154, 2356–2370. doi: 10.1099/mic.0.2008/019539-0, PMID: 18667568

[ref38] Schumacher M. A. den Hengst C. D. Bush M. J. Le T. B. K. Tran N. T. Chandra G.. (2018). The MerR-like protein BldC binds DNA direct repeats as cooperative multimers to regulate *Streptomyces* development. Nat. Commun. 9:1139. doi: 10.1038/s41467-018-03576-3, PMID: 29556010 PMC5859096

[ref39] Sevcikova B. Kormanec J. (2003). The ssgB gene, encoding a member of the regulon of stress-response sigma factor sigmaH, is essential for aerial mycelium septation in *Streptomyces coelicolor* A3(2). Arch. Microbiol. 180, 380–384. doi: 10.1007/s00203-003-0603-y, PMID: 14513208

[ref40] Smirnov A. Esnault C. Prigent M. Holland I. B. Virolle M. J. (2015). Phosphate homeostasis in conditions of phosphate proficiency and limitation in the wild type and the phoP mutant of *Streptomyces lividans*. PLoS One 10:e126221. doi: 10.1371/journal.pone.0126221, PMID: 25978423 PMC4433243

[ref41] Sola-Landa A. Rodriguez-Garcia A. Amin R. Wohlleben W. Martin J. F. (2013). Competition between the GlnR and PhoP regulators for the glnA and amtB promoters in *Streptomyces coelicolor*. Nucleic Acids Res. 41, 1767–1782. doi: 10.1093/nar/gks1203, PMID: 23248009 PMC3561978

[ref42] Sola-Landa A. Rodriguez-Garcia A. Apel A. K. Martin J. F. (2008). Target genes and structure of the direct repeats in the DNA-binding sequences of the response regulator PhoP in *Streptomyces coelicolor*. Nucleic Acids Res. 36, 1358–1368. doi: 10.1093/nar/gkm1150, PMID: 18187507 PMC2275107

[ref43] Sola-Landa A. Rodriguez-Garcia A. Franco-Dominguez E. Martin J. F. (2005). Binding of PhoP to promoters of phosphate-regulated genes in *Streptomyces coelicolor*: identification of PHO boxes. Mol. Microbiol. 56, 1373–1385. doi: 10.1111/j.1365-2958.2005.04631.x, PMID: 15882427

[ref44] Tiwari S. Da C. M. Almeida S. Hassan S. S. Jamal S. B. Oliveira A.. (2014). C. Pseudotuberculosis phop confers virulence and may be targeted by natural compounds. Integr. Biol. 6, 1088–1099. doi: 10.1039/c4ib00140k 25212181

[ref45] Tu J. Huang B. Zhang Y. Zhang Y. Xue T. Li S.. (2016). Modulation of virulence genes by the two-component system PhoP-PhoQ in avian pathogenic *Escherichia coli*. Pol. J. Vet. Sci. 19, 31–40. doi: 10.1515/pjvs-2016-0005, PMID: 27096785

[ref46] Walters S. B. Dubnau E. Kolesnikova I. Laval F. Daffe M. Smith I. (2006). The *Mycobacterium tuberculosis* PhoPR two-component system regulates genes essential for virulence and complex lipid biosynthesis. Mol. Microbiol. 60, 312–330. doi: 10.1111/j.1365-2958.2006.05102.x, PMID: 16573683

[ref47] Wang X. Wei J. Xiao Y. Luan S. Ning X. Bai L. (2021). Efflux identification and engineering for ansamitocin P-3 production in *Actinosynnema pretiosum*. Appl. Microbiol. Biotechnol. 105, 695–706. doi: 10.1007/s00253-020-11044-6, PMID: 33394151

[ref48] Wei C. Ding T. Chang C. Yu C. Li X. Liu Q. (2019). Global regulator PhoP is necessary for motility, biofilm formation, exoenzyme production and virulence of *Xanthomonas citri* Subsp. citri on citrus plants. Gene 10:340. doi: 10.3390/genes10050340, PMID: 31064142 PMC6562643

[ref49] Wu Y. Kang Q. Zhang L. L. Bai L. (2020). Subtilisin-involved morphology engineering for improved antibiotic production in actinomycetes. Biomol. Ther. 10:851. doi: 10.3390/biom10060851, PMID: 32503302 PMC7356834

[ref50] Yang R. Liu X. Wen Y. Song Y. Chen Z. Li J. (2015). The PhoP transcription factor negatively regulates avermectin biosynthesis in *Streptomyces avermitilis*. Appl. Microbiol. Biotechnol. 99, 10547–10557. doi: 10.1007/s00253-015-6921-6, PMID: 26298701

[ref51] Yin L. Li Q. Xue M. Wang Z. Tu J. Song X.. (2019). The role of the phoP transcriptional regulator on biofilm formation of avian pathogenic *Escherichia coli*. Avian Pathol. 48, 362–370. doi: 10.1080/03079457.2019.1605147, PMID: 30958690

[ref52] Yu T. W. Bai L. Clade D. Hoffmann D. Toelzer S. Trinh K. Q.. (2002). The biosynthetic gene cluster of the maytansinoid antitumor agent ansamitocin from *Actinosynnema pretiosum*. Proc. Natl. Acad. Sci. U. S. A. 99, 7968–7973. doi: 10.1073/pnas.092697199, PMID: 12060743 PMC123004

[ref53] Zhong C. Zong G. Qian S. Liu M. Fu J. Zhang P.. (2019). Complete genome sequence of *Actinosynnema pretiosum* x47, an industrial strain that produces the antibiotic ansamitocin AP-3. Curr. Microbiol. 76, 954–958. doi: 10.1007/s00284-018-1521-1, PMID: 29858620

[ref54] Zhou L. Lei X. H. Bochner B. R. Wanner B. L. (2003). Phenotype microarray analysis of *Escherichia coli* K-12 mutants with deletions of all two-component systems. J. Bacteriol. 185, 4956–4972. doi: 10.1128/JB.185.16.4956-4972.2003, PMID: 12897016 PMC166450

