# Supplementary material for: Deletion of the Response Regulator PhoP Accelerates the Formation of Aerial Mycelium and Spores in Actinosynnema pretiosum
Source: Front Microbiol. 2022 Apr 6;13:845620. doi: 10.3389/fmicb.2022.845620 (PMC9019756; doi:10.3389/fmicb.2022.845620)
Supplement: Supplementary file 1 [file Data_Sheet_1.docx]

**Supplementary Material**

TABLE S1 Primers used in this study

| **Primers** | **Description or sequence (5'-3')** |
| --- | --- |
| PhoP-L-F | ACCCGGGGATCCTCTAGAGATTTCTAGAACCACGCGGGTCTCCTCG |
| PhoP-L-R | GATGGAGCTGCACATGAACCGGTGATACGAAGACGCTGGAC |
| PhoP-R-F | TCCGACTAACGAAAATCCCGGAGCATCAGGTCGAGCAGG |
| PhoP-R-R | GCATGCCTGCAGGTCGACGATAAAGCTTGCTTACGATGCGTGGGTG |
| Apra-F | CCGGTTCCGCTCAGCCAATCGACTGGCGAG |
| Apra-R | AGGGGCGACAATGTCATCAGCGGTGGAGTG |
| PhoP-V-F | AACTTGTAGCCCAGACCTCG |
| PhoP-V-R | GCTGTGACCAGGGTGCTCAT |
| PhoP-Com-F | ACCCGGGGATCCTCTAGAGATTAAGCTTACAGGTGGACCGTGTTGGA |
| PhoP-Com-R | GCATGCCTGCAGGTCGACGATAAAGCTTTTCTCCCCGCTCCAGCAC |
| PhoP-pET15b F | TGCCGCGCGGCAGCCATATGACCAGGGTGCTCATCGTG |
| PhoP-pET15b R | TTAGCAGCCGGATCCTCGAGTCAGGACTCGAACTTGTAGC |
| ssgB F | TCTTTCCCTACACGACTCGTCGTTACCCTCTTCT |
| ssgB R | GTGACTGGAGTTCCTTTTCGCGCAACAGCCGGTA |
| flaD F | TCTTTCCCTACACGACTGTGATTTCCTCCGTGAATC |
| flaD R | GTGACTGGAGTTCCTTCCCCAGAGCCCGCCCTTC |
| flg B F | TCTTTCCCTACACGACCGGCTCGTGCTCCCTATGCC |
| flg B R | GTGACTGGAGTTCCTTCGGCAACCGCGCCCCGGTTGA |
| cheA F | TCTTTCCCTACACGACCAGGCTCCCGCCATGTCGAA |
| cheA R | GTGACTGGAGTTCCTTCCTGCCCATCACCCGGCTC |
| FliM F | TCTTTCCCTACACGACCGCCGCCTAGTACGGCCGT |
| FliM R | GTGACTGGAGTTCCTTACCCCGATCCGGTGACCGG |
| swrD F | TCTTTCCCTACACGACCGGCTTCCGTCCTTGGAT |
| swrD R | GTGACTGGAGTTCCTTACGGGTGACGGGCGGCCG |
| whiB F | TCTTTCCCTACACGACCCTGCGCGGGATCACCTTGA |
| whiB R | GTGACTGGAGTTCCTTTCGAGACCGCCTCCTTGGC |
| Biotin F | Biotin-TCTTTCCCTACACGAC |
| Biotin R | Biotin-GTGACTGGAGTTCCTT |
| hrdB-realtime-F | AGCCCGAGGACATGGAAG |
| hrdB-realtime-R | CCAGACGAAGTCGCCCTC |
| phoP-realtime-F | GTCCAGCGTCTTCGTATCACC |
| phoP-realtime-R | CGACCTGCTGGAGTACCTC |
| phoR-realtime-F | CCTCCTGCATGATCTTCGC |
| phoR-realtime-R | AGCTCAAGACCCCGGTGGGC |

TABLE S2 Putative target genes of PhoP in *A. pretiosum*

| **Gene locus** | **Gene product** | **Sequences** | **Distance to start**  **codon** | **Score** | **Homologous genes in *S. coelicolor*** |
| --- | --- | --- | --- | --- | --- |
| CNX65_RS00080 | hypothetical protein | GTTCACCC | -150 | 8.2 |  |
| CNX65_RS00120 | penicillin-binding protein | GTTCATTT | -2 | 8.8 |  |
| CNX65_RS00195 | hypothetical protein | GTTCACTC | -25 | 8.5 |  |
| CNX65_RS00200 | helix-turn-helix domain-containing protein | GTTCACTC | -200 | 8.5 |  |
| CNX65_RS00200 | helix-turn-helix domain-containing protein | AATTGAAC | -2 | 8.8 |  |
| CNX65_RS00215 | hypothetical protein | GACTGAAC | -93 | 7.6 |  |
| CNX65_RS00255 | KR domain-containing protein | GTTCAGCC | -52 | 7.3 |  |
| CNX65_RS00265 | hypothetical protein | GTTCACCC | -71 | 8.2 |  |
| CNX65_RS00270 | hypothetical protein | GCATGAAC | -40 | 8.2 |  |
| CNX65_RS00295 | hypothetical protein | GTTCACGC | -2 | 8.2 |  |
| CNX65_RS00305 | class I SAM-dependent methyltransferase | GTGTGAAC | -63 | 8.5 |  |
| CNX65_RS00310 | hypothetical protein | GTTCATCC | -89 | 8.2 |  |
| CNX65_RS00310 | hypothetical protein | GGCTGAAC | 5 | 7.3 |  |
| CNX65_RS00335 | DUF983 domain-containing protein | GTTCACCC | -79 | 8.2 |  |
| CNX65_RS00345 | S49 family peptidase | GGTTGAAC | -53 | 8.2 |  |
| CNX65_RS00365 | DUF485 domain-containing protein | GTTCACAC | -227 | 8.5 |  |
| CNX65_RS00365 | DUF485 domain-containing protein | GTTCATGC | -45 | 8.2 |  |
| CNX65_RS00370 | cation acetate symporter | GCGTGAAC | -2 | 8.2 | SCO1822 |
| CNX65_RS00385 | glycerophosphodiester phosphodiesterase | GTTCACCC | -63 | 8.2 |  |
| CNX65_RS00405 | DUF952 domain-containing protein | GTTCATCC | -35 | 8.2 |  |
| CNX65_RS00430 | CPBP family intramembrane metalloprotease | GCGTGAAC | -37 | 8.2 |  |
| CNX65_RS00465 | cytochrome ubiquinol oxidase subunit I | GTGTGAAC | -2 | 8.5 | SCO3945 |
| CNX65_RS00680 | DUF2587 domain-containing protein | GTTCATGC | -2 | 8.2 |  |
| CNX65_RS00705 | N-acetyltransferase | GCATGAAC | -2 | 8.2 |  |
| CNX65_RS00720 | bifunctional metallophosphatase/5'-nucleotidase | AGCTGAAC | -171 | 7.6 |  |
| CNX65_RS00725 | MarR family transcriptional regulator | AGCTGAAC | -31 | 7.6 |  |
| CNX65_RS00740 | histidinol-phosphate transaminase | GTTCACCC | -101 | 8.2 |  |
| CNX65_RS00745 | carboxymethylenebutenolidase | GTTCACCC | -28 | 8.2 |  |
| CNX65_RS00830 | antitoxin | GCGTGAAC | -124 | 8.2 |  |
| CNX65_RS00830 | antitoxin | GAGTGAAC | -2 | 8.5 |  |
| CNX65_RS00880 | hypothetical protein | GTTCACCC | -52 | 8.2 |  |
| CNX65_RS00900 | hypothetical protein | ACGTGAAC | -2 | 8.5 |  |
| CNX65_RS00970 | ABC transporter permease | GGTTGAAC | -10 | 8.2 |  |
| CNX65_RS00990 | membrane-associated protein | ACCTGAAC | -57 | 7.6 |  |
| CNX65_RS01045 | organic hydroperoxide resistance protein | GCATGAAC | -2 | 8.2 |  |
| CNX65_RS01075 | hypothetical protein | GTTCACCC | -108 | 8.2 |  |
| CNX65_RS01095 | tRNA-Pro | AGGTGAAC | -20 | 8.5 |  |
| CNX65_RS01100 | metallophosphoesterase | GTTCACCT | -2 | 8.5 |  |
| CNX65_RS01110 | PASTA domain-containing protein | GTGTGAAC | -240 | 8.5 |  |
| CNX65_RS01115 | WhiB family transcriptional regulator | GTGTGAAC | -22 | 8.5 | SCO3579 |
| CNX65_RS01190 | MFS transporter | GGGTGAAC | -188 | 8.2 |  |
| CNX65_RS01190 | MFS transporter | GGCTGAAC | -167 | 7.3 |  |
| CNX65_RS01220 | serine protease | GGGTGAAC | -2 | 8.2 |  |
| CNX65_RS01240 | acetate--CoA ligase | GTTCAGAC | -83 | 7.6 |  |
| CNX65_RS01275 | MurR/RpiR family transcriptional regulator | GTTCACCC | -113 | 8.2 |  |
| CNX65_RS01290 | beta-N-acetylhexosaminidase | GGGTGAAC | -158 | 8.2 | SCO2758 |
| CNX65_RS01295 | HAD-IB family hydrolase | GACTGAAC | -195 | 7.6 | SCO5558 |
| CNX65_RS01295 | HAD-IB family hydrolase | GTTCAGTC | -176 | 7.6 |  |
| CNX65_RS01300 | hypothetical protein | GTTCAGTC | -237 | 7.6 |  |
| CNX65_RS01300 | hypothetical protein | GACTGAAC | -218 | 7.6 |  |
| CNX65_RS01405 | hypothetical protein | GTTCACGC | -2 | 8.2 |  |
| CNX65_RS01410 | hypothetical protein | GTTCAACC | -182 | 8.2 |  |
| CNX65_RS01410 | hypothetical protein | GTTCACGC | -48 | 8.2 |  |
| CNX65_RS01455 | DNA primase | GTTCAAGT | -79 | 8.5 |  |
| CNX65_RS01520 | tRNA-Thr | GTTCACTC | 27 | 8.5 |  |
| CNX65_RS01590 | coenzyme F420 biosynthesis-associated protein | GTGTGAAC | -11 | 8.5 |  |
| CNX65_RS01605 | VWA domain-containing protein | GTTCAACC | -230 | 8.2 |  |
| CNX65_RS01610 | ATP-dependent metallopeptidase FtsH/Yme1/Tma family protein | GTTCAACC | -243 | 8.2 | SCO3404 |
| CNX65_RS01805 | metal ABC transporter substrate-binding protein | AACTGAAC | -56 | 7.9 |  |
| CNX65_RS01850 | cysteine--tRNA ligase | GGGTGAAC | -39 | 8.2 |  |
| CNX65_RS01900 | trehalose-phosphatase | AGCTGAAC | -48 | 7.6 |  |
| CNX65_RS01900 | trehalose-phosphatase | GTTCAGCT | -44 | 7.6 |  |
| CNX65_RS01905 | trehalose-6-phosphate synthase | GTTCACCT | -102 | 8.5 |  |
| CNX65_RS01935 | VWA domain-containing protein | GTTCACCC | -196 | 8.2 |  |
| CNX65_RS01970 | ADP-ribosylglycohydrolase family protein | ACCTGAAC | -134 | 7.6 |  |
| CNX65_RS01975 | SARP family transcriptional regulator | ACCTGAAC | -45 | 7.6 |  |
| CNX65_RS02050 | FadR family transcriptional regulator | GGGTGAAC | -28 | 8.2 |  |
| CNX65_RS02080 | threonine/serine dehydratase | GTTCACTT | -79 | 8.8 |  |
| CNX65_RS02120 | 3-oxoacyl-ACP reductase | GGGTGAAC | -8 | 8.2 |  |
| CNX65_RS02125 | TetR/AcrR family transcriptional regulator | GGGTGAAC | -21 | 8.2 |  |
| CNX65_RS02155 | phosphomethylpyrimidine synthase ThiC | GTTCAGGC | -42 | 7.3 | SCO3928 |
| CNX65_RS02200 | hypothetical protein | AGGTGAAC | -2 | 8.5 |  |
| CNX65_RS02260 | copper homeostasis protein CutC | GAGTGAAC | -103 | 8.5 | SCO4315 |
| CNX65_RS02310 | hypothetical protein | GGGTGAAC | -48 | 8.2 |  |
| CNX65_RS02345 | hypothetical protein | GTTCAGAC | -54 | 7.6 |  |
| CNX65_RS02380 | LysM peptidoglycan-binding domain-containing protein | GTTCAGGC | -124 | 7.3 |  |
| CNX65_RS02530 | DUF2537 domain-containing protein | GCCTGAAC | -2 | 7.3 |  |
| CNX65_RS04530 | type I glutamate--ammonia ligase GlnA | AGATGAAC | 29 | 8.5 | SCO2198 |
| CNX65_RS33700 | phosphate transport system regulatory protein PhoU | GTTCATCC | -61 | 8.2 | SCO4228 |
| CNX65_RS33730 | phosphate ABC transporter substrate-binding protein PstS | AGGTGAAC | -111 | 8.5 | SCO4142 |
| CNX65_RS33730 | phosphate ABC transporter substrate-binding protein PstS | ACATGAAC | -100 | 8.5 |  |
| CNX65_RS33730 | phosphate ABC transporter substrate-binding protein PstS | GATTGAAC | -34 | 8.5 |  |


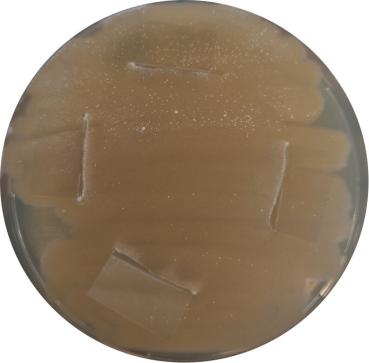


**Figure S1.** The picture of the device used for sample preparation for SEM analysis.


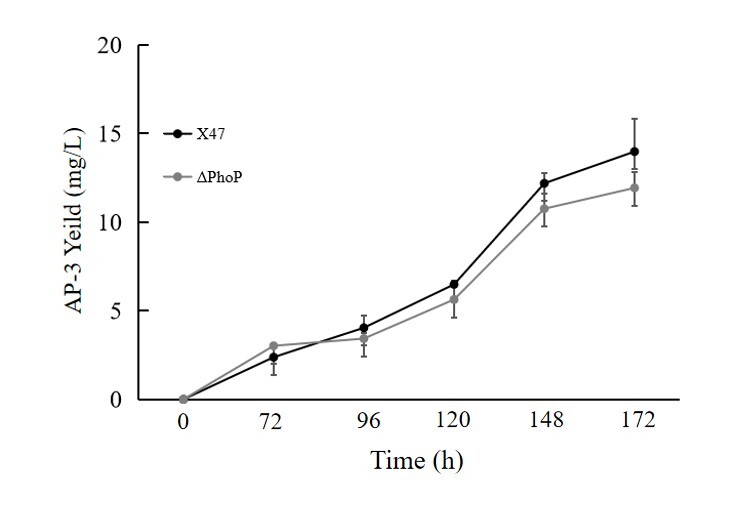


**Figure S2.**  HPLC analysis of AP-3 production in the X47 strain and ∆phoP.


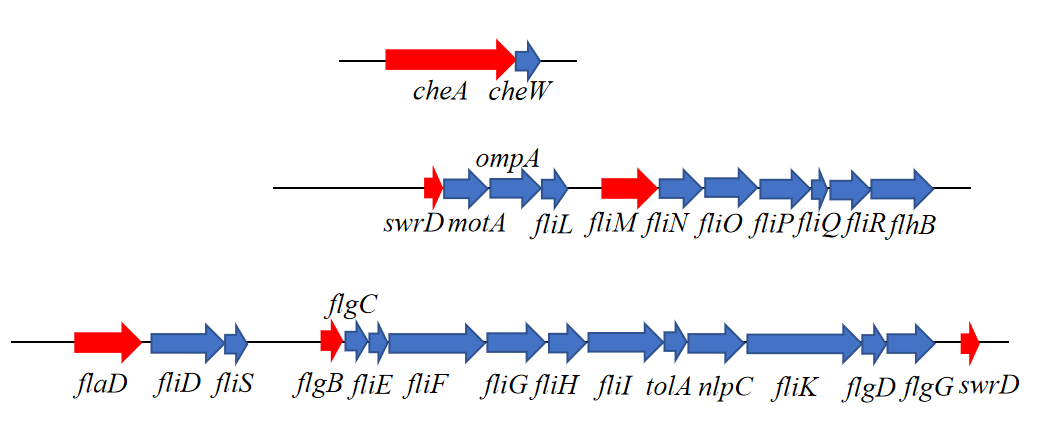


**Figure S3.** The arrangements of genes related to flagellum biosynthesis and motility. Red arrows indicate the first gene of each putative operon.


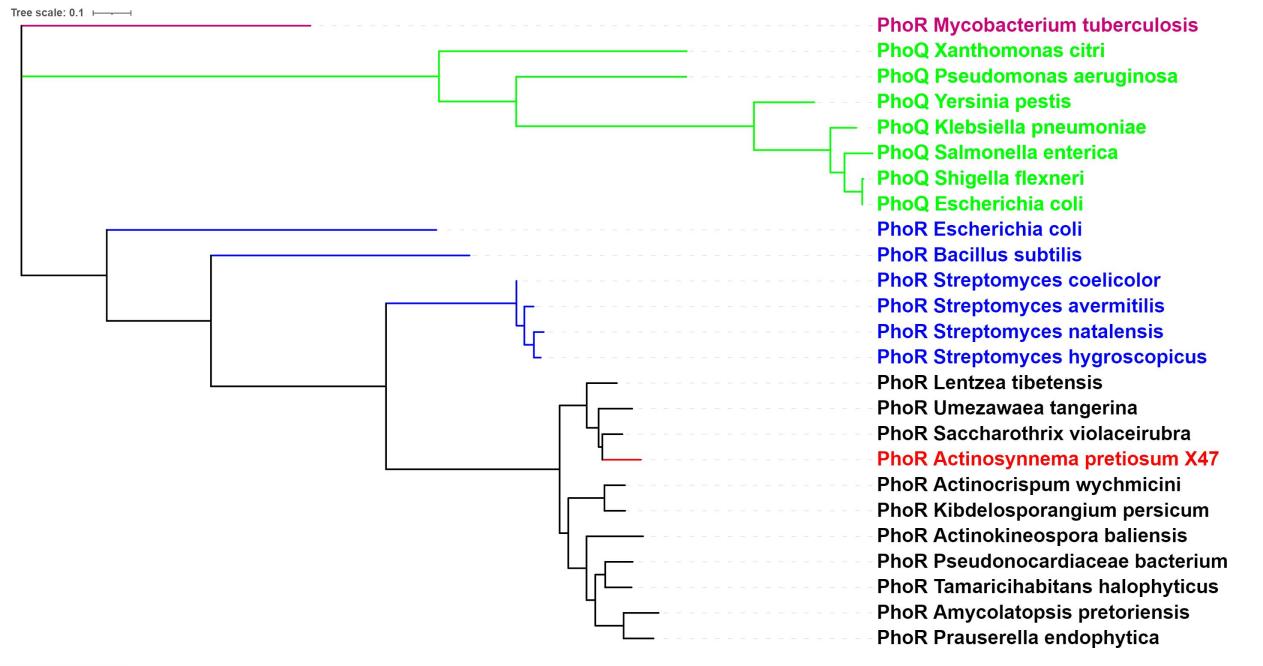


**Figure S4.** Phylogenetic tree analysis of PhoR.

## Antimony molybdenum blue spectrophotometry

The concentration of phosphate in the medium was analyzed by antimony molybdenum blue spectrophotometry method. Ascorbic acid solution was added into ISP2 liquid medium and fully mixed. Molybdate solution was then added into the mixture, and make the volume up to 50 mL by adding distilled water. Leave the solution for 15 min at room temperature, and the absorbance was measured at 700 nM. The standard curve was established by phosphate standard solution (2.0 µg/L).
